# The analgesic and anti-inflammatory effects of zukamu granules, a traditional Chinese medical formulation

**DOI:** 10.1080/13880209.2019.1675716

**Published:** 2019-12-03

**Authors:** Qiang Yin, Shuaihong Hou, Hailong Yin, Dandan Mu, Dajun Jiang, Fang Tian, Jing Jing Li, Fang Huang

**Affiliations:** a Department of Basic Medicine, Hubei University of Chinese Medicine, Wuhan, PR China;; b Xinjiang Uygur Pharmaceutical Co., Ltd, Wulumuqi, PR China;; c Ministry of Education, Key Laboratory of Traditional Chinese Medicine Resource and Prescription, Wuhan, PR China

**Keywords:** Inflammatory cytokines, NF-κB, lung injury, pharmacodynamic

## Abstract

**Context:** Zukamu granule, a traditional Chinese medicine, has shown clinical treatment efficacy. However, the pharmacodynamic effects and possible anti-inflammatory mechanisms of zukamu are still unclear.

**Objective:** To investigate the analgesic and anti-inflammatory effects and possible mechanisms of zukamu granules on acute lung injury.

**Materials and methods:** Kunming mice and Sprague Dawley rats were gavaged with zukamu (1.35, 2.7 and 5.4 g/kg, respectively) or ganmaoling (GMLG; 2.7 g/kg) once a day for 7 d. Dexamethasone treatment (5 mg/kg) were administered only on the last day. Analgesic effects were evaluated through the hot plate test and acetic acid writhing test. The expression of cytokines and proteins was measured in serum and lung tissues to elucidate the efficacy of zukamu against lung injury.

**Results:** Significant analgesic effects were observed at 30 min after zukamu administration at medium and high doses (*p* < 0.05), but the effect was not obvious at low dose until 60 min post-administration (*p* > 0.05). Zukamu treatment at all doses notably reduced the lung wet-to-dry (W/D) ratios compared to that of model rats (*p* < 0.05) and the effect was more evident at high dose compared to those at medium and low doses. The levels of cytokines and proteins in the lung tissues were inhibited by zukamu.

**Conclusions:** Zukamu exhibited analgesic and protective effects against lung injury *via* regulating NF-κB signalling and inflammatory cytokines. As zukamu granules contain multiple ingredients, further exploration of the mechanisms underlying its analgesic and anti-inflammatory functions were needed.

## Introduction

Influenza and common colds, induced by viral infection, are the most common respiratory diseases worldwide. They exert a negative impact on the upper respiratory organs, such as the throat and lungs, and other organs, such as the brain and muscles (Moghadami 2017). The symptoms are characterized by headaches, high fever, sore throats, coughing and runny noses, with occasional pulmonary complications, such as pneumonia, in severe cases of influenza. In general, the clinical symptoms of the common cold are relatively mild (Eccles 2007). One way or another, both the common cold and influenza caused by upper respiratory infection are the main reasons of seeking medical care for children, elderly adults or immunocompromized individuals (Santee et al. 2016). According to health care providers, the expenses of treatment, quality of life and effects on work and school undoubtedly cause social burdens (Santee et al. 2016; Wang, Jiang et al. 2017).

Despite improvements in the efficacy of western medicine for the treatment of the common cold, there is still no established medication that targets pathogens to cure a cold (Wang, Jiang et al. 2017). The therapeutic efficacy of western medicine is based on improving the symptoms and inhibiting the occurrence of complications using analgesics, antitussives and antipyretics (Picon et al. 2013). There is also a broad spectrum of antiviral drugs, but they are limited in application to clinical treatments due to drug resistance. Adverse reactions of western medication occasionally appear, such as dizziness, drowsiness and gastrointestinal discomfort (Dudek et al. 2010). Drug resistance and toxic side effects on other organs may also occur after long-term use (Bower et al. 2007; Liu et al. 2014).

Traditional Chinese medicine has been widely applied in the treatment of diseases for thousands of years in China, especially in the treatment of headaches and common cold. The therapeutic effects have been increasingly obvious and traditional Chinese medicine has progressively gained popularity around the world (Lou et al. 2015; Chen et al. 2016). Compounds used in traditional Chinese medicine usually involve complex compositions of different materials. Thus, their therapeutic efficacy is the synergistic result of multiple compounds from corresponding plants or herbs.

Zukamu granule is a formula in Chinese medicine from the Xinjiang Uygur Autonomous Region in China, composed of the resurrection lily rhizome (*Kaempferia galanga* Linn. [Zingiberaceae]), pygmy water lily (*Nymphaea tetragona* Georgi [Nymphaeaceae]), pobumuguo (*Cordia dichotoma* Forst. [Boraginaceae]), mentha (*Mentha haplocalyx* Briq. [Labiatae]), jujube (*Ziziphus jujuba* Mill. [Rhamnaceae]), manzanilla (*Matricaria recutita* Linn. [Compositae]), liquorice (*Glycyrrhiza uralensis* Fisch. [Leguminosae]), seed of hollyhock (*Althaea rosea* (L.) Cav. [Malvaceae]), *Rheum officinale* Baill. (Polygonaceae) and poppy capsule (*Papaver somniferum* L. [Papaveraceae]). The compound was manufactured by modern science and technology based on a secret ancient prescription from Uygur. In the past, the prescription was the first choice for local physicians to cure common colds. Although the efficacy of zukamu granules has been confirmed in clinical practice for the treatment of the common cold or upper respiratory infection in the Xinjiang area in China (Liu et al. 2014; Xing et al. 2015), the pharmacological and pharmacodynamic properties of zukamu, especially its mechanism of action, have not been adequately investigated.

This study was designed to examine the pharmacodynamic effects of zukamu granules based on animal models with the intention of exploring their possible anti-inflammatory mechanisms. The results of this study will provide a theoretical basis of the efficacy and analgesic and anti-inflammatory effects of zukamu and promote the clinical application of zukamu in China and in other countries.

## Materials and methods

### Chemicals and materials

Zukamu granules were purchased from Xinjiang Uygur Pharmaceutical Co., Ltd. (Xinjiang, China). Ganmaoling granules (GMLG) were acquired from Sanjiu Medical & Pharmaceutical Co., Ltd. (Shenzhen, China). Lipopolysaccharides (LPS), dexamethasone (DEX) and acetic acid were purchased from Sigma-Aldrich Inc. (St. Louis, MO, USA). Primary and secondary antibodies were acquired from Abcam (Cambridge, UK), Cell Signalling Technology (Boston, MA, USA) and Bioswamp (Wuhan, China). Haematoxylin and eosin were obtained from Bioswamp (Wuhan, China). TRIzol reagent and M-MLV reserve transcriptase were acquired from Invitrogen (Carlsbad, CA, USA).

### Animals

Kunming mice and Sprague–Dawley rats were purchased from Hubei provincial centre for disease control and prevention (Wuhan, China). Adult Kunming mice weighing 20 ± 5 g and Sprague Dawley rats weighing 200 ± 25 g were housed in controlled conditions (temperature, 22 ± 2 °C and relative humidity 50 ± 2%) with free access to water and food in a 12 h dark/light cycle. The analgesic effect of zukamu was assessed in mice, whereas the acute lung injury model was constructed in rats. All animal experiments were performed in accordance with the requirements of the Ethics of Animal Experiments and had been approved by the Animal Experimental Ethical Inspection of Laboratory Animal Centre, Huazhong Agriculture University (No.HZAUMO-2017-034).

### Drug treatment

Zukamu is widely used in the clinical treatment and the clinical dose is 36 g/d. As the mouse is used in this experiment, the equivalent dose of the drug is 5.4 g/kg according to the body surface area method (Xu 2002), and this dose is taken as the high dose group of Zukamu; ¼ and ½ of the equivalent dose are used as low and middle dose of Zukamu, respectively. After adaptive feeding for a week, the mice and rats were treated with zukamu at low, medium and high dose (1.35, 2.7 and 5.4 g/kg, respectively). As positive controls, mice were treated with 2.7 g/kg GMLG (positive analgesic) by intragastric administration and rats were treated with 5 mg/kg DEX (positive anti-inflammatory agent) by intraperitoneal injection. The mice and rats in the control (no treatment) and model (acute lung injury using LPS, rats only) groups were gavaged with the same amount of physiological saline. Zukamu and GMLG were administered once a day for seven days. For DEX treatment, the drug was administered only on the last day.

### Hot plate test

Female mice were subjected to a hot plate testing to evaluate physical pain after drug administration. The temperature of the plate was continuously monitored and controlled at 55 ± 0.1 °C. Pre-selection of non-treated mice for the test was performed by placing each mouse on the hot plate and recording the time (in seconds) at which the mouse licked its right hind paw as a pain threshold. If the pain threshold was above 30 s, the mouse was eliminated. The remaining eligible mice were subjected to hot plate testing, which was conducted at 30, 60 and 90 min after treatment with drugs or physiological saline on the last day. The determination of pain threshold followed the same steps as those for pre-selection. If the mouse did not lick its paws within 60 s, it was removed from the plate to avoid burns and the pain threshold was recorded as 60 s.

### Acetic acid writhing test

Male mice were subjected to acetic acid writhing tests to evaluate acute chemical pain after drug administration. All mice were placed in the experimental environment at least 2 h before acetic acid administration. Mice were intraperitoneally injected with 0.1 mL/10 g of 0.6% acetic acid solution (Naghizadeh et al. 2016) 60 min after the administration of drugs or physiological saline on the last day. The male mice were observed for concave abdomen, extension of trunks and hind limbs and lifting of buttocks, indicating abdominal writhing. The number of abdominal writhes was calculated within 20 min, starting at 5 min after the injection of acetic acid solution.

### Acute lung injury model and measurement of wet-to-dry (W/D) ratio of lung weight

The acute lung injury model was established in male rats by LPS administration (5 mg/kg, intravenous injection) (Shen et al. 2009) and the rats were sacrificed 6 h after LPS injection. The lungs were collected quickly and the left lung was separated. The wet weight of the left lung was measured after simple handling with filter paper. The left lung was then dried at 60 °C for 48 h and the dry weight of the lung was measured to calculate the W/D ratio.

### Haematoxylin-eosin staining and pathological observation

The collected right lung was directly fixed in 4% neutral formaldehyde for 48 h and subjected to conventional dehydration, transparentizing, dipping and embedding. Paraffin-embedded tissue blocks were sliced into 5 μm thick sections. The sections were stained with haematoxylin and eosin solution and the pathological morphology of the lung tissues was observed with optical microscopy.

### Detection of inflammatory cytokines in serum

The serum levels of tumour necrosis factor (TNF)-α, interleukin (IL)-1β, IL-12 and IL-6 were detected using enzyme-linked immunosorbent assay kits (Bioswamp, China) based on the manufacturer’s instructions. All experiments were conducted in triplicate.

### Western blot

The expression levels of proteins related to the nuclear factor kappa-light-chain-enhancer of activated B cells (NF-κB) signalling pathway in the lung tissues were examined by western blot. Part of the right lung tissue was homogenized and lysed in immunoprecipitation assay buffer (Solarbio Life Sciences, China) after washing with Tris-buffered saline (TBS). Total proteins were collected by high-speed centrifugation and quantitatively analysed by the bicinchoninic acid method. The proteins (20 μg) were subjected to sodium dodecyl sulphate-polyacrylamide gel electrophoresis and transferred to polyvinylidene fluoride membranes. Then the membranes were incubated with 5% skim milk in TBS at room temperature for 1.5 h. After washing with TBS/Tween, the membranes were incubated overnight at 4 °C with the following primary antibodies: NF-κB p65 (ab16502, 1:1000), p-NF-κB p65 (ab28856, 1:1000), IκBα (ab32518, 1:1000), cyclooxygenase-2 (COX-2, ab15191, 1:1000), inducible nitric oxide synthase (iNOS, ab3523, 1:1000) and GAPDH (#2118, 1:1000). Second antibody incubation was performed for 1 h at room temperature using goat anti-rabbit IgG (PAB160011, 1:10000) conjugated with horseradish peroxidase. Finally, the membranes were treated with enhanced chemiluminescence reagent and imaged with an automatic chemical analyser (Tanon Science & Technology Co., Ltd., China). The grey scale of protein expression was analysed by ImageJ software and normalized to GAPDH.

### Quantitative reverse transcription polymerase chain reaction (qRT-PCR)

The lung tissues were ground with liquid nitrogen and total RNA was extracted by TRIzol reagent. The total RNA was quantified and reverse transcription was performed using M-MLV reverse transcriptase. Quantitative reverse transcription polymerase chain reaction **(**qRT-PCR) was conducted as described previously (Kanda et al. 2017) with the following primers: COX-2 F, 5′-GGGTGTCCCTTCGCCTCTTT-3′, R, 5′-GTTGCCGGTATCT GCCTCA-3′; iNOS F, 5′-TGTGCTAATGCGGAAGGTCAT-3′, R, 5′-CGACTTTCCTGTCTCAGTAGCAAA-3′; GAPDH F, 5′-CCA GGGCTGCCTTCTCTTGT-3′, R, 5′-CCAGCCTTCTCCATGGT GGT-3′ (Kanda et al. 2017).

### Statistical analysis

Statistical analysis was conducted with SPSS version 22.0 statistical software. The data were expressed as the mean ± standard deviation (SD). Data between groups were compared by one-way analysis of variance with least significant difference multiple comparison test. *p* < 0.05 indicates statistical significance.

## Results

### Zukamu is an effective analgesic

**Figure 1.**
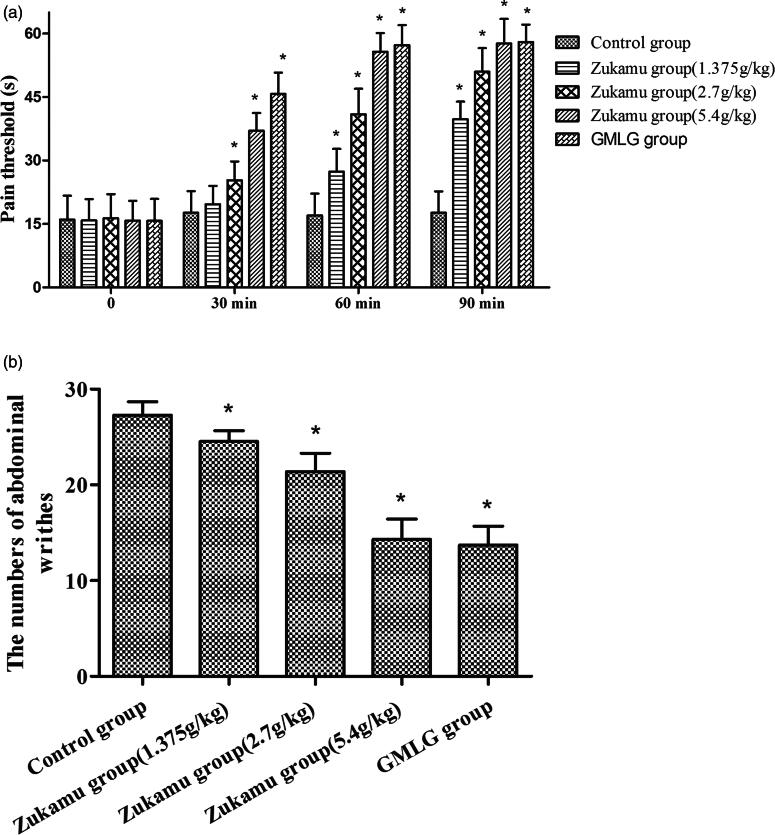
Analgesic effects of zukamu in mice. Kunming strain mice were treated with different doses of zukamu (1.35, 2.7 and 5.4 g/kg) and positive analgesic control drug GMLG by intragastric administration. The analgesic effects against physical and chemical pain induced by hot stimulation and acetic acid, respectively, were measured by (a) hot plate test and (b) acetic acid writhing test. The data represent the mean ± SD, *n* = 10 per group. *p< 0.05 *vs.* control.

The analgesic effect of zukamu was measured by hot plate and acetic acid writhing testing in Kunming mice. Zukamu relieved the physical and chemical pain induced by heat stimulation and acetic acid, respectively. The pain threshold of control mice remained constant at different detection points (Figure 1(A)). In each individual zukamu dose group, the pain threshold increased with detection time after drug administration, whereas for GMLG, the pain threshold increased up to 60 min. Significant analgesic effect was observed at 30 min after zukamu administration at medium and high doses, but the effect was not obvious at low dose until 60 min post-administration. Comparison between dose groups suggested that the analgesic effect was significantly augmented with the increase in concentration. However, no predominant changes were observed between high-dose zukamu and GMLG administration until 60 min after treatment. These results indicated that zukamu exerted a time- and dose-dependent analgesic effect to ease physical pain.

Inflammatory pain was inflicted upon mice by intraperitoneal injection of acetic acid after treatment with physiological saline, zukamu or GMLG, a positive analgesic control drug. The acetic acid writhing test showed that the numbers of abdominal writhes in mice treated with zukamu at all doses were reduced to higher extents with increasing dose (*p*< 0.05, Figure 1(b)). These results displayed the dose-dependent analgesic effect of zukamu.

### Protective effect of zukamu on LPS-induced acute lung injury in rats

#### Zukamu decreased the lung W/D ratio of rats with acute lung injury

**Figure 2.**
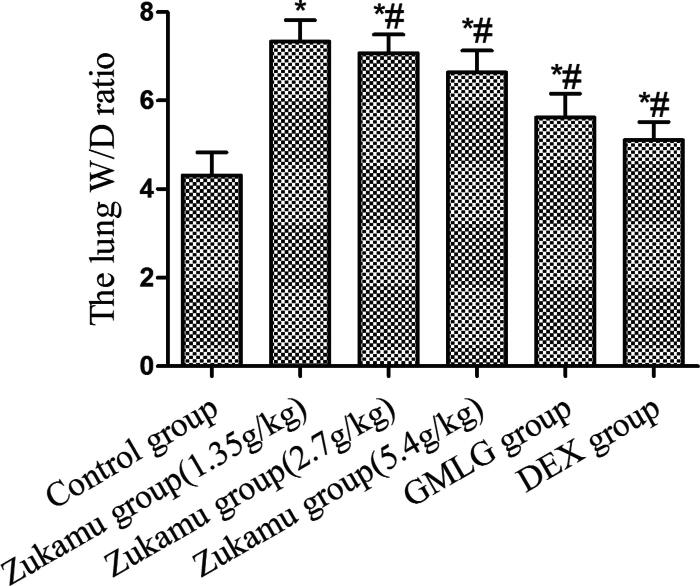
Zukamu decreased the lung W/D ratio of rats with acute lung injury. Sprague–Dawley rats were treated with different doses of zukamu (1.35, 2.7 and 5.4 g/kg) (ig.) and positive anti-inflammatory control drug DEX (ip.) Acute lung injury was induced by LPS injection 60 min after drug administration on the last day. The left lung tissues were collected, the wet and dry weights of the lungs were measured, and the lung W/D ratios were calculated. The data represent the mean ± SD, *n* = 10 per group. *p< 0.05 *vs.* control, ^#^*p*< 0.05 *vs.* Model.

The rat model of acute lung injury was constructed *via* tail vein injection of LPS and the degree of lung injury was evaluated by measuring the W/D ratio of the left lung. As shown in Figure 2, LPS-induced acute lung injury (Model) caused the W/D ratio to increase significantly compared to that of control rats (*p*< 0.05). Zukamu treatment at all doses notably reduced the lung W/D ratios compared to that of model rats (*p*< 0.05), and the effect was more evident at high dose compared to those at medium and low doses of zukamu. The results implicate that zukamu ameliorated acute lung injury in rats, as characterized by the reduction in lung W/D ratio.

#### Zukamu ameliorated the pathological morphology of acute lung injury in rats

**Figure 3.**
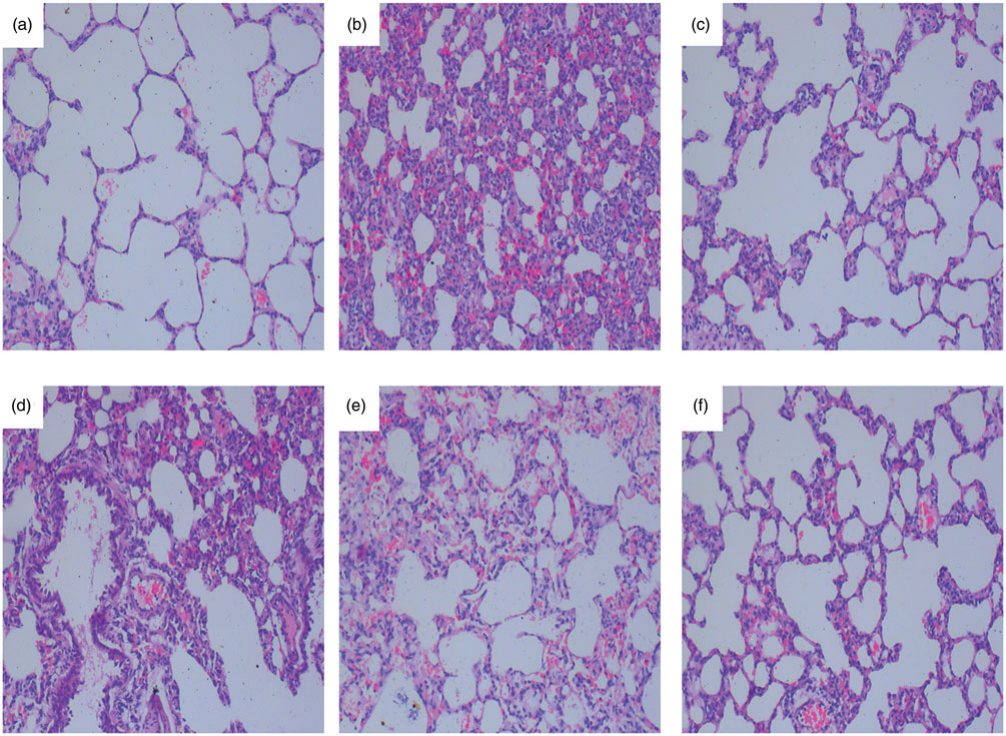
Effect of zukamu on the pathological morphology in rats (200×). Haematoxylin-eosin staining of the pathological morphology of right lung tissues in (a) control rats, (b) model rats, and rats treated by (c) DEX, (d) low-dose zukamu, (e) medium-dose zukamu and (f) high-dose zukamu.

Normal lung tissue morphology is shown in Figure 3(a) and typical histopathological changes indicating acute lung injury were observed after LPS-induced modelling (Figure 3b). The signs involve alveolar haemorrhage, thickened alveolar walls, broadened and oedematous alveolar septa, and a large number of infiltrated inflammatory cells. Administration of zukamu, especially at high dose, and GMLG effectively improved the constitution of the lung and decreased inflammatory cell infiltration (Figure 3(c–f)). These macroscopic observations signify the curative and protective effects of zukamu against acute lung injury.

#### Zukamu reduced serum levels of inflammatory cytokines in rats with acute lung injury

**Figure 4.**
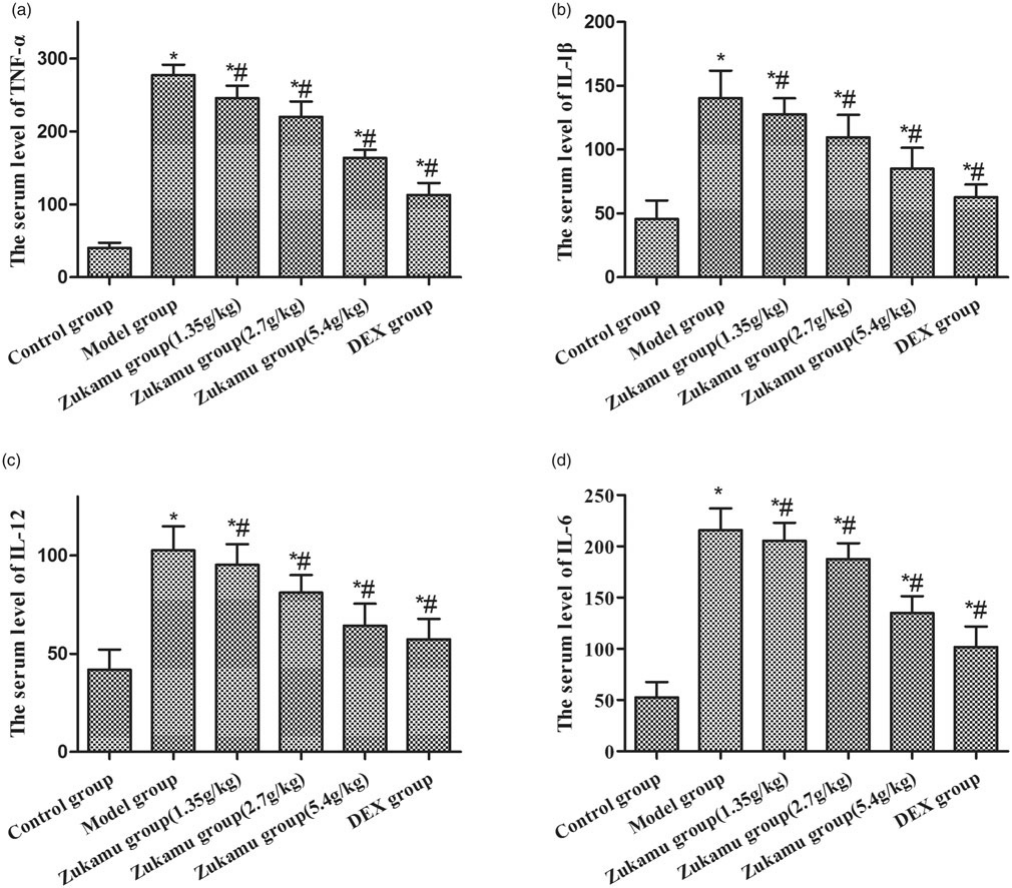
Serum expression of (a) TNF-α, (b) IL-1β, (c) IL-12 and (d) IL-6. The data represent the mean ± SD, *n* = 5–8 per group. *p< 0.05 *vs.* control, ^#^*p*< 0.05 *vs.* Model.

The serum levels of inflammatory cytokines were measured and analysed 6 h after LPS treatment. Rats with acute lung injury (Model) showed elevated levels of TNF-α (Figure 4(a)), IL-1β (Figure 4(b)), IL-12 (Figure 4(c)) and IL-6 (Figure 4(d)) (*p*< 0.05), indicating the onset of inflammatory response. The serum expression of these cytokines was markedly suppressed by zukamu at all doses and by DEX treatment (*p* < 0.05), signifying that inflammation was relieved.

#### Zukamu inhibited the activity of NF-κB signalling pathway in lung tissue

**Figure 5.**
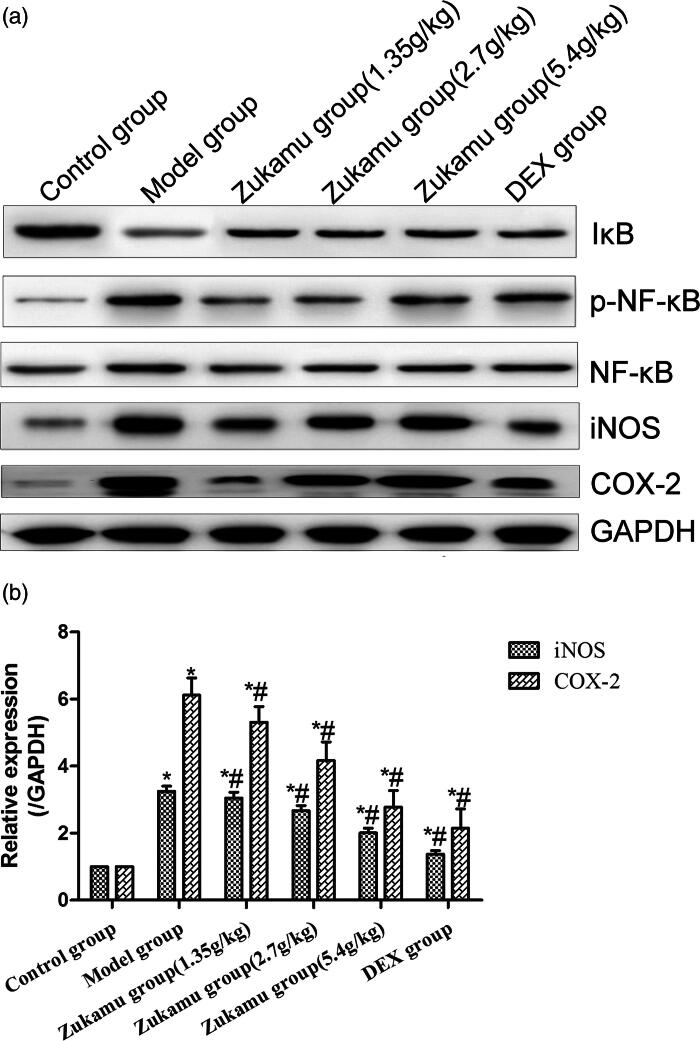
Regulation of NF-κB signalling pathway by zukamu. (a) Expression of proteins related to NF-κB signalling was detected by western blot. (b) mRNA levels of COX-2 and iNOS were evaluated by qRT-PCR. The data represent the mean ± SD, *n* = 3 per group. *p< 0.05 *vs.* control, ^#^*p*< 0.05 *vs.* Model.

The NF-κB signalling pathway plays an important role in regulating the expression of inflammatory cytokines. Thus, the expression levels of NF-κB and proteins related to NF-κB signalling in lung tissues were analysed after LPS challenge. LPS reduced the expression of IκB and induced the phosphorylation of NF-κB in lung tissues (Figure 5(a)), and treatment with zukamu or DEX reversed these effects. In addition, the expression of COX-2 and iNOS was enhanced after LPS administration, and these effects were markedly suppressed by zukamu and DEX (Figure 5(a,b)).

## Discussion

Traditional Chinese medicine has been studied seriously and applied to treat patients with the common cold, and its good curative effects in the clinical treatment of common cold have been proven. GMLG, a type of Chinese compound medicine, is widely used to treat patients with symptoms of influenza or common cold such as headaches, sore throats, stuffy noses and fever (Mao et al. 2004; Lou et al. 2015). Composed of a variety of chemicals, GMLG exerts physiological functions including fever reduction, pain relief and bacterial and viral inhibition (Mao et al. 2004).

Zukamu, a compound formula composed of ten materials, was developed based on a secret ancient prescription from Uygur and has been used clinically in the Xinjiang Uygur Autonomous Region in China for many years. The majority of studies has focussed on evaluating the clinical efficacy of zukamu by data analysis. Thus, the pharmacological properties of zukamu, especially its mechanism of action, are inadequately investigated.

This study was designed to explore the analgesic and anti-inflammatory effects of zukamu and its possible mechanisms, using GMLG as a positive analgesic control drug. Experimental animals exposed to zukamu were subjected to the hot plate test to determine the effects of the drug on pain relief.

The observations suggested that different doses of zukamu relieved pain to varying degrees, as the pain thresholds increased with increasing zukamu dose. The maximal analgesic activity of zukamu was noted at 60 min after the drug administration, after which the effect of the drug was maintained at a constant level. The analgesic effect of zukamu was confirmed again by the acetic acid writhing test. Collectively, the tests showed that zukamu is an effective analgesic against both physical and chemical pain.

Influenza viral infection and bacterial infection can cause inflammatory responses in the upper respiratory tract, which in turn triggers lung injury. The model of acute lung injury was established to evaluate the anti-inflammatory activity of zukamu. Examination of the lung W/D ratio and pathologic morphology provided evidence of the anti-inflammatory and protective effect of zukamu against LPS-induced acute lung injury in rats.

The activation of inflammation-related signalling pathways, including NF-κB and p38/mitogen-activated protein kinase pathways (Wang, Zhou et al. 2017), triggers inflammatory reactions in influenza. As a nuclear transcription factor, NF-κB plays an essential role in facilitating cell proliferation, apoptosis and inflammatory response by regulating the expression of growth factors, cytokines, interferons, transcription factors and chemokines (Pahl 1999). The influenza virus induces excessive immune response and overexpression of cytokines and chemokines, regulated by NF-κB signalling (Chen et al. 2015), and the replication of the influenza virus could be suppressed by inhibiting the activity of NF-κB (Dudek et al. 2010; Ramos and Fernandez-Sesma 2015). In this study, LPS-induced increases in NF-κB phosphorylation were inhibited by zukamu, leading to the upregulation of IκB and indicating that zukamu modulated the anti-inflammatory effect by inhibiting NF-κB signalling.

In addition, inflammatory cytokines such as TNF-α and IL-6 are multifunctional and related to lung damage during inflammation induced by pathogenic infection (Chen et al. 2015). The overexpression of TNF-α, IL-1β, IL-12 and IL-6 can mediate inflammatory responses via the NF-κB signalling pathway, and pro-inflammatory cytokines can also lead to the overexpression of other inflammatory mediators, such as iNOS and COX-2 (Pérez-Sala and Lamas 2001; Karin et al. 2006). The expression of COX-2 was reported to be directly regulated by the p65 subunit of NF-κB binding to the COX-2 promoter (Ulivi et al. 2008). This study showed that the levels of inflammatory cytokines TNF-α, IL-1β, IL-12 and IL-6 were enhanced after LPS challenge and subsequently reduced by zukamu. At the same time, the LPS-induced increase in COX-2 and iNOS expression in lung tissues was inhibited by zukamu, especially at high dose. Therefore, it can be concluded that zukamu suppressed inflammatory responses by reducing cytokine levels.

## Conclusions

Taken together, zukamu has analgesic and anti-inflammatory effects against pain and acute lung injury. The anti-inflammatory effect was achieved by reducing the expression of inflammatory cytokines and inhibiting the NF-κB signalling pathway. Zukamu granules contain multiple ingredients and exert a variety of pharmacological functions, but the other activities of this compound medicine and of the specific active ingredients (for example, which ingredients are responsible for anti-inflammation and analgesia) remain unclear. It is necessary to further explore the active compounds of zukamu and the mechanisms underlying its analgesic and anti-inflammatory functions.
